# Whole genome sequencing of three native chicken varieties (Common Deshi, Hilly and Naked Neck) of Bangladesh

**DOI:** 10.1038/s41597-024-04291-z

**Published:** 2024-12-24

**Authors:** Md Ataul Goni Rabbani, Adriana Vallejo-Trujillo, Zhou Wu, Katarzyna Miedzinska, Shakila Faruque, Kellie A. Watson, Jacqueline Smith

**Affiliations:** 1 The Roslin Institute and Royal (Dick) School of Veterinary Studies, The University of Edinburgh, Easter Bush Campus, Midlothian, EH25 9RG UK; 2 Poultry Production Research Division, Bangladesh Livestock Research Institute (BLRI), Savar, Dhaka, 1341 Bangladesh

**Keywords:** Next-generation sequencing, DNA sequencing

## Abstract

Bangladeshi indigenous chicken varieties - Common Deshi, Hilly and Naked Neck are notable for their egg production, meat quality, extraordinary survivability and disease resistance. However, the potential to harness their unique genetic merits are being eroded by various factors, including crossbreeding. In-depth genomic studies have not been carried out on these breeds so far. To this end, blood samples and associated phenotypic metadata have been collected from local, unimproved birds sampled from 8 different locations across the country, and from Bangladesh Livestock Research Institute (BLRI)-improved chickens of the same mentioned breeds. Whole Genome Sequencing (WGS) of 96 selected samples, representing local and improved populations of each breed, has been carried out. Around 22 M high-quality SNPs have been identified, with 25% of these being novel variants previously undescribed in public databases. This data set will allow for genetic comparison between breeds, and between selected and unimproved birds, providing a resource for genomic selection in Bangladeshi breeding schemes to create more productive and resilient poultry stock.

## Background & Summary

There are several native chicken varieties in Bangladesh of which Common Deshi (CD) or Non-descript Deshi, Hilly (HL) and Naked Neck (NN) chickens are noteworthy for their egg production, meat quality and survivability in harsh environmental conditions. Consumers prefer indigenous chicken meat and eggs due to their special characteristics of smell, taste and texture^1,2^. Some studies found that around 98% of consumers pay attention to particular qualities such as fat content, meat colour and taste, eggshell colour, size and yolk colour^3^. Domestic local chicken is thus preferred over intensively produced commercial hybrid chicken in Bangladesh.

However, these precious chicken populations have been undergoing genetic erosion since the introduction of improved stocks (both pure lines of different chicken breeds and commercial hybrids) from developed countries. This has occurred as a result of various factors like the incorporation of exotic chicken breeds and commercial hybrids, indiscriminate cross-breeding, sub-optimal breeding strategies and lack of conservation programmes^2,4^. Recognising the potential of Bangladeshi local chicken varieties, the Bangladesh Livestock Research Institute (BLRI) started a conservation and improvement programme for the three above-mentioned native chicken varieties around 2011^5^, applying conventional breeding strategies. Both egg production and growth performance have improved significantly as compared to the foundation stock^6–8^. These native chickens maintained by the BLRI are known as ‘BLRI Improved Native Chicken’. However, apart from a few studies using RAPD markers, microsatellite markers or partial mitochondrial DNA-loop sequences to understand the maternal origin^9–12^, advanced genomic research on these native breeds has yet to be conducted. In-depth genome-level research on these promising native chicken species will be crucial for the identification of potential genomic regions and candidate genes responsible for productivity improvement, disease resistance and stress tolerance potential, to harvest maximum utilization. In addition, appropriate breeding strategies based on genomic surveys need to be undertaken for the conservation of native chicken germplasms.

Advances in genomics have enabled Whole Genome Sequencing (WGS), allowing scientists to uncover genomic insights, contributing to livestock breeding and development^13–19^. For instance, WGS analysis of 234 indigenous African chickens identified around 15 million SNPs, of which 14% represent unique variants, with some being associated with environmental adaptation and other important traits^20^.

In this article, we report whole-genome sequencing data from 96 Bangladeshi native chickens. The samples include BLRI improved native chickens and the same variety of local chickens from eight different locations across the country. The indigenous chicken samples taken from various villages in Bangladesh are referred to here as ‘Unimproved Native Chicken’. Paired-end next-generation sequencing for short reads was carried out on all samples with an average 23X coverage and reads mapped to the GRCg7b chicken reference genome (GCA_016699485.1; https://ftp.ensembl.org/pub/release-109/fasta/gallus_gallus/dna/). More than 22 million biallelic Single Nucleotide Polymorphisms (SNPs) were identified in this study, with 25% being novel variants.

The utility of data generated from the present study is expected to include helping evaluate genetic variation and diversity of Bangladeshi native chicken varieties, detecting important genomic regions and candidate genes underlying different economic traits of interest (for example, egg production, body weight and stress tolerance ability) and future investigation on genomic selection as a potential strategy in chicken breeding programmes. The data can also help make associations between the genome and the environment using Ecological Niche Modelling (ENM)^21–25^ and aid in the development of SNP chips/imputation panels^26–30^. This is the first-time whole genome sequencing from Bangladeshi native chicken varieties has been presented, at scale, thus providing a valuable resource for avian researchers to understand the genetics of local chickens. These WGS data will also help to enrich the efforts of the Chicken Genomic Diversity Consortium^31^ to reveal origins and adaptations of global chicken populations.

## Methods

### Sampling locations

**Fig. 1:**
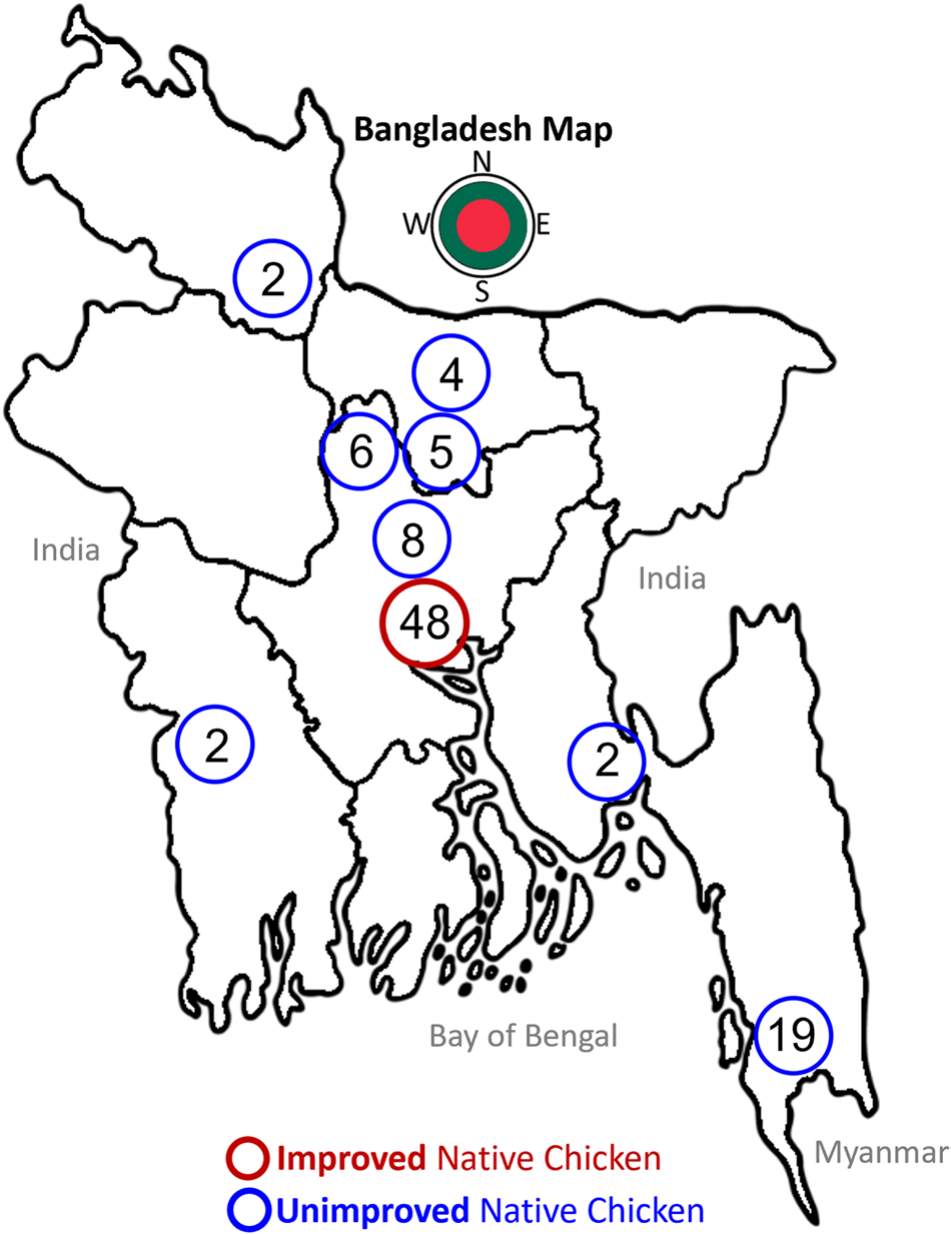
Sample collection sites from across Bangladesh. Blue circles indicate unimproved native chicken sampling locations (across the different villages) while the red circle indicates the improved native chicken sampling location (BLRI Headquarters, Dhaka, Bangladesh). The numbers inside the circles indicate the number of samples that were sequenced from each location.

390 blood samples were collected from birds from different geographical locations in Bangladesh (Fig. 1) between February and June 2022. Of the total samples, 215 blood samples were taken from BLRI-improved chickens represented by three native varieties (Common Deshi, Hilly and Naked Neck) at the BLRI Poultry Research Farm. 175 blood samples were also taken from unimproved indigenous chickens (of the same three breeds) from several villages in eight different geographical locations across the country. While selecting the sampling sites, different environmental or climatic factors like temperature and humidity variation and availability of native chicken variety in that particular region, were taken into consideration. Details of the collected samples are shown in Fig. 1 and described in Supplementary Table 1.

### Ethical approval

All relevant ethical approvals were obtained from the Animal Experimentation Ethics Committee (AEEC) of BLRI [AEEC/BLRI00116]. In addition, prior collection of blood samples from the village chickens was done with the full consent of the owners. The samples were transported to the United Kingdom under a material transfer agreement (MTA) between BLRI and The University of Edinburgh (MTA Roslin 3324). In addition, import authorization for blood samples was issued under the Trade in Animals and Related Products (Scotland) Regulations (2012).

### Blood collection

Around 0.5 ml blood was withdrawn from the brachial wing vein (or cutaneous ulnar vein) of the selected healthy chickens using 1.0 ml insulin syringe (JMI Syringes & Medical Devices Limited, Bangladesh) following the procedure stated by Kelly and Alworth^32^. Chickens were handled with extreme care, maintaining standard animal handling procedures to ensure minimum stress. Immediately after collection, the blood was gently and carefully spread onto Whatman FTA classic cards (Cat. No: WHAWB120205; Merck, Germany). After completion of blood collection, the FTA cards were kept at room temperature for at least three hours to allow complete drying. Appropriate caution was taken to keep the FTA cards safe from direct sunlight. All air-dried blood samples were then stored safely in double zipper bags until further processing at the Roslin Institute (Edinburgh, UK).

### Sample selection for WGS

For whole genome sequencing, a total of 96 samples were selected from the 390 samples collected. The selected samples consisted of equal numbers of male and female chickens from each of the 6 groups (3 improved and 3 unimproved indigenous chicken varieties) as shown in Table 1. In the case of selecting BLRI-improved native chickens, unrelated chickens were given priority, considering the pedigree data (parental information) of the previous two generations of the sampled chicken populations. Samples from all geographical locations were included while selecting unimproved chickens for WGS. Again, within the same sampling area, individuals were selected from different villages.

**Table 1:** Selected samples (total = 96) for WGS from BLRI improved native chicken (n = 48) and unimproved native chickens from local villages (n = 48).

Population Type	Chicken Variety	Sampling location	Number of samples	
			Male	Female
BLRI-Improved chickens (n = 48)	Common Deshi (CD)	BLRI HQ*, Savar, Dhaka	8	8
	Hilly (HL)	BLRI HQ, Savar, Dhaka	8	8
	Naked Neck (NN)	BLRI HQ, Savar, Dhaka	8	8
	**A. BLRI-Improved sub-total =**		**24**	**24**
Unimproved chickens (n = 48)	Common Deshi (CD)	Monirumpur, Jessore	1	1
		Dhamrai, Dhaka	2	2
		Sadar, Mymensingh	1	1
		Bhaluka, Mymensingh	1	1
		Chhagalnaiya, Feni	1	1
		Gopalpur, Tangail	1	1
		Sadar, Gaibandha	1	1
		**a**. Sub-total of Unimproved CD =	8	8
	Hilly (HL)	Naikhongchhari, Bandarban	5	5
		BLRI Regional Research Farm, Naiknongchhari, Bandarban	3	3
		**b**. Sub-total of Unimproved HL =	8	8
	Naked Neck (NN)	Dhamrai, Dhaka	2	2
		Naikhongchhari, Bandarban	2	1
		Sadar, Mymensingh	0	2
		Bhaluka, Mymensingh	2	1
		Gopalpur, Tangail	2	2
		**c**. Sub-total of Unimproved NN =	8	8
	**B. Unimproved sub-total (a + b + c) =**		**24**	**24**
**Total (A + B)=**			**48**	**48**

^*^Poultry Research Farm, BLRI Headquarters, Savar, Dhaka-1341, Bangladesh.

### Sample preparation for WGS

All the selected blood samples on FTA cards were processed using a QIAamp® DNA Investigator-50 kit (CAT no: 56504; QIAGEN, Germany). The quality and the quantity of the isolated genomic DNA were assessed by three different instruments - NanoDrop 1000 spectrophotometer (Thermo Fisher Scientific, USA), Qubit 4 fluorometer (Invitrogen, USA) and TapeStation 4200 (Agilent Technologies, USA). After normalising to a final volume of 50 μL, the genomic DNA samples were sent to BGI Genomics, Poland for whole genome sequencing (150 bp paired-end, 20X coverage).

### Library preparation and sequencing

Library preparation was conducted at BGI Genomics in Poland. Before sequencing, concentration, integrity and purity of all the genomic DNA samples were again checked. Concentration was determined by Qubit Fluorometer (Invitrogen) and sample integrity and purity were detected by Agarose Gel Electrophoresis (concentration of agarose gel: 1%, voltage: 150 V, electrophoresis time: 40 minutes). Random fragmentation of genomic DNA was done using a Covaris (E220) instrument, then the fragmented genomic DNA was selected by Magnetic beads [Cat no: 1000005278 (MGI Easy DNA Clean Beads; https://en.mgi-tech.com/Products/reagents_info/id/7] to an average size of 200–400 bp. Fragments were then end repaired and 3′ adenylated, with adaptors then ligated to the ends of these 3′ adenylated fragments. PCR was then carried out to amplify the fragments with adaptors and PCR products were purified by magnetic beads. The double stranded PCR products were then denatured and circularised by the splint oligo sequence. The single strand circular DNA (ssCir DNA) was formatted as the final library. The library was then assessed by quality control. The library was amplified with phi29 to make DNA nanoballs (DNB) which have more than 300 copies of each molecule. The DNBs were loaded into the patterned nanoarray and paired end 150 bp reads were generated by combinational Probe-Anchor Synthesis (cPAS). The sequencing was performed using the next generation high-throughput platform at BGI Genomics (DNBSEQ-T7) in paired-end mode (~20X coverage).

### Data processing

**Fig. 2:**
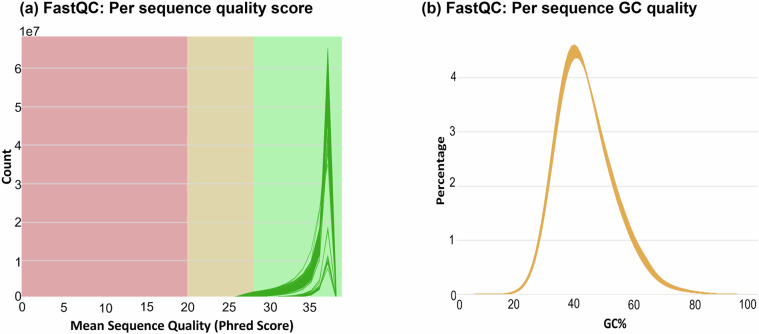
Quality control metrics from FastQC analysis. (**a**) Per sequence quality scores and (**b**) per sequence GC content.

Raw reads were filtered using a series of data processing steps to remove adaptor sequences, contamination and low-quality reads. This was done using SOAPnuke software^33^ by BGI Genomics. Upon receipt of sequence data, quality of the sequences was checked using the FastQC programme (version 0.11.7)^34^. For ease of reviewing the sequence quality, FASTQC reports for all 96 samples were aggregated in a single report by the MultiQC (version 1.1) package^35^, examples from which are shown in Fig. 2. No adaptor sequences were present and as the quality of the raw reads was very high, no further quality-based trimming was performed on the sequence reads.

**Fig. 3:**
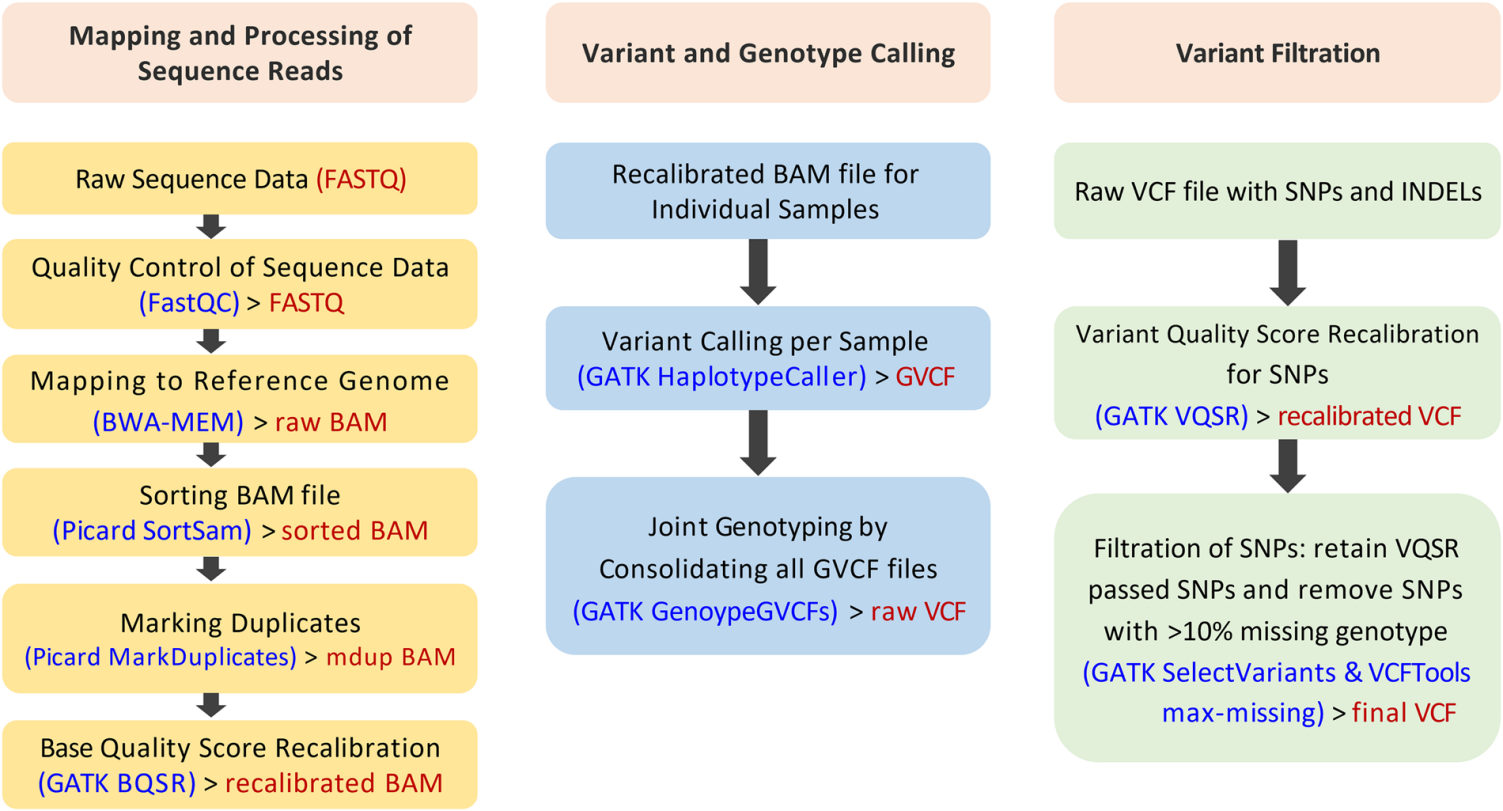
Overview of the sequence alignment, variant calling and variant filtration process.

Mapping of the sequence reads was performed against the GRCg7b chicken reference genome (https://www.ebi.ac.uk/ena/browser/view/GCA_016699485.1) using Burrows-Wheeler Aligner (bwa-version 0.7.15)^36,37^ with default parameters. Before alignment, sequence dictionary and FASTA index files were created using Samtools (version 1.13)^38^ which were used by the BWA-MEM programme. The resultant Sequence Alignment Map (SAM) files then underwent some further processing steps such as sorting according to their coordinates using the SortSam programme of Picard tools (version 2.25.4)^39^ and marking duplicate reads using the MarkDuplicate programme of the same tool. BAM files were then validated to troubleshoot errors such as improper formatting, faulty alignments and incorrect flag values. In addition, different WGS metrics were calculated using both Samtools and Picard tools. Base Quality Score Recalibration (BQSR) was then carried out using the BaseRecalibrator tool from the Genome Analysis Toolkit - GATK (version 4.0.10.1)^40,41^ to correct the biases in the quality scores assigned by the sequencer. The final recalibrated BAM files were then used for further downstream analysis. The overview of the mapping and variant calling steps is presented in Fig. 3.

GATK best practice guidelines for germline short variant discovery were followed for variant calling and SNP detection using the HaplotypeCaller tool with the ‘-ERC GVCF’ settings to generate GVCF files which then underwent joint genotyping using the GenomicsDBImport and then GenotypeGVCFs functions. The Variant Quality Score Recalibration (VQSR)^42^ function was then applied to perform variant filtration using a set of around one million validated SNPs^26^ as a training and true set, with over 21 M chicken SNPs from the Ensembl database (release-110)^43^ used as known variants.

The following annotations or context statistics were considered during the VQSR step: read depth (DP), variant quality by depth (QD), root mean square mapping quality (MQ), mapping quality rank sum test statistics (MQRankSum), read position rank sum test statistics (ReadPosRankSum), and strand bias statistics (FS and SOR). A tranche sensitivity threshold of 99% was applied for filtering variants. As the final quality control of the called variants, any SNPs with a missing genotype rate more than 10% across the samples were filtered out using VCFtools^44^ (version 0.1.13).

All codes used for the mapping and variant calling steps are included in the Supplementary materials and also available on GitHub.

## Data Records

All full-length raw sequencing data in FASTQ format can be accessed from the Sequence Read Archive (SRA) of the NCBI database under BioProject accession number PRJNA1027325^45^. The filtered VCF file containing more than 22 million high-quality autosomal biallelic SNPs can also be accessed from the European Necleotide Archive (ENA) and the European Variation Archive (EVA) repositories under the Project accession number PRJEB78357^46,47^ and Analysis accession number ERZ24818048.

## Technical Validation

### Quality control of sequencing data

The total number of bases generated from the sequencing each sample was from 24 Gb to 28 Gb, with GC content averaging 42%. Around 95.33% of the bases had a minimum Phred scaled quality score of 30 which indicates a base calling accuracy of 99.9%. The average estimated genome coverage across all sample was ~23X (after marking duplicate reads) with the range varying from 20X to 25X. FastQC reports (shown in Fig. 2) indicate that sequencing quality of all samples was of high-quality. The average mapping rate of the sequence reads against the reference genome was 99.60%, which further confirmed the high quality of the sequencing data.

#### Quality control of SNP data

During the variant calling step using GATK best practice guidelines, more than 30 M total variants were identified including more than 26 M SNPs and 4.5 M insertions/deletions (INDELs). VQSR filtering was then applied to ensure identification of high-quality variants and to minimize the number of false positives. More than 1 M validated SNPs^26^ and about 22 M SNPs from Ensembl^43^ were used as known variants (training data set) during the VQSR step. The VQSR filtering retained 100% of the SNPs (26.07 M). Next, only the biallelic SNPs (22.75 M) were taken into consideration for downstream analysis which included a further filtering step. SNPs with a missing genotype rate of more than 10% were discarded, which retained around 22 M high-quality SNPs. In this step, minimum genotype quality (GQ) score was considered 20 (–minGQ 20.0), depth of sequence coverage was 3 (–minDP 3), Hardy-Weinberg Equilibrium (HWE) value was 0.00001 (–hwe 0.00001) along with the maximum missing rate of genotypes of 10% (–max-missing 0.9) using VCFtools. Annotation of the identified SNPs was done using Ensembl’s Variant Effect Predictor-VEP^48^ (release-110) which revealed that around 75% of the total high-quality variants are already reported in the public databases, but the rest were novel variants. We found that the majority of the identified variants (around 68%) were intronic while around 2% were exonic variants (shown in Table 2a). Again, we found a greater number of variants in unimproved chicken populations compared to improved chickens of the same varieties (Table 2b). Details of SNPs in different annotation categories in both improved and unimproved native chicken populations are given in Supplementary Table 2.

**Table 2:** (**a**) SNPs in different annotation categories for overall Bangladeshi native chicken populations. (**b**) Sumary statistics of SNPs in Bangladeshi improved and unimproved native chicken populations.

Variant Categories		Variant Counts	Variants %	% of Total	
**(a)**					
**Exonic Variants**	Synonymous variant	232,772	1.050	1.702	
	Missense variant	140,947	0.636		
	Stop-gained variant	2,040	0.009		
	Stop-lost variant	246	0.001		
	Start-lost variant	1,050	0.005		
	Stop-retained variant	171	0.001		
	Coding sequence variant	1	0.000		
**Splicing Variants**	Splice acceptor variant	1,925	0.009	0.468	
	Splice donor variant	2,733	0.012		
	Splice donor 5^th^ base variant	3,120	0.014		
	Splice region variant	46,812	0.211		
	Splice donor region variant	9,705	0.044		
	Splice polypyrimidine tract variant	39,445	0.178		
**Intronic Variants**	Intron variant	15,111,660	68.181	68.181	
**Intergenic Variants**	Intergenic variant	2,964,795	13.377	13.377	
**Regulatory Variants**	Upstream gene variant	1,342,998	6.059	9.828	
	Downstream gene variant	835,260	3.769		
**UTR variants**	5 prime UTR variant	144,292	0.651	2.789	
	3 prime UTR variant	473,793	2.138		
**Other variants**	Mature miRNA variant	230	0.001	3.655	
	Non-coding transcript exon variant	809,920	3.654		
**Total = **		**22,163,915**	**100.00**	**100.00**	
**(b)**					
**Chicken populations**	**Total SNPs**	**Novel SNPs**	**Novel SNPs %**	**Ts/Tv Ratio**	**Singletons (%)**
CD_Imp	14,585,310	1,646,054	11.29	2.482	16.57
CD_Unimp	17,142,624	2,677,720	15.62	2.475	23.61
HL_Imp	13,792,070	1,493,434	10.83	2.485	14.8
HL_Unimp	15,873,233	2,257,509	14.22	2.479	20.84
NN_Imp	11,889,880	992,111	8.34	2.492	12.52
NN_Unimp	17,041,300	2,632,800	15.45	2.475	23.11
Overall Bangladeshi native chicken population	22,163,915	5,553,897	25.1	2.453	13.64

CD = Common Deshi, HL = Hilly, NN = Naked Neck, Imp = BLRI Improved native chickens and Unimp=Unimproved native chickens from different localities of Bangladesh.

To determine the quality of SNP calling from the high-throughput sequencing data, the transition/transversion ratio (Ts/Tv) can be employed. We obtained a transition/transversion ratio (Ts/Tv) of 2.453 in the overall populations which is typically found to be ~2 for whole genome sequence data^49^. Some previous studies reported a Ts/Tv ratio between 2.17 to 2.69 for different indigenous chicken breeds^20,50–53^. Unless it is too high (>4), a higher Ts/Tv ratio generally indicates better SNP calling^54^.

The proportion of singleton SNPs in the overall population for this study was 13.64% while the unimproved chicken populations have higher percentages compared to the improved populations (Table 2b). These reflect the genetic diversity between the studied chicken populations. In addition, smaller sample size may be also responsible for a large proportion of singleton SNPs^55^.

**Fig. 4:**
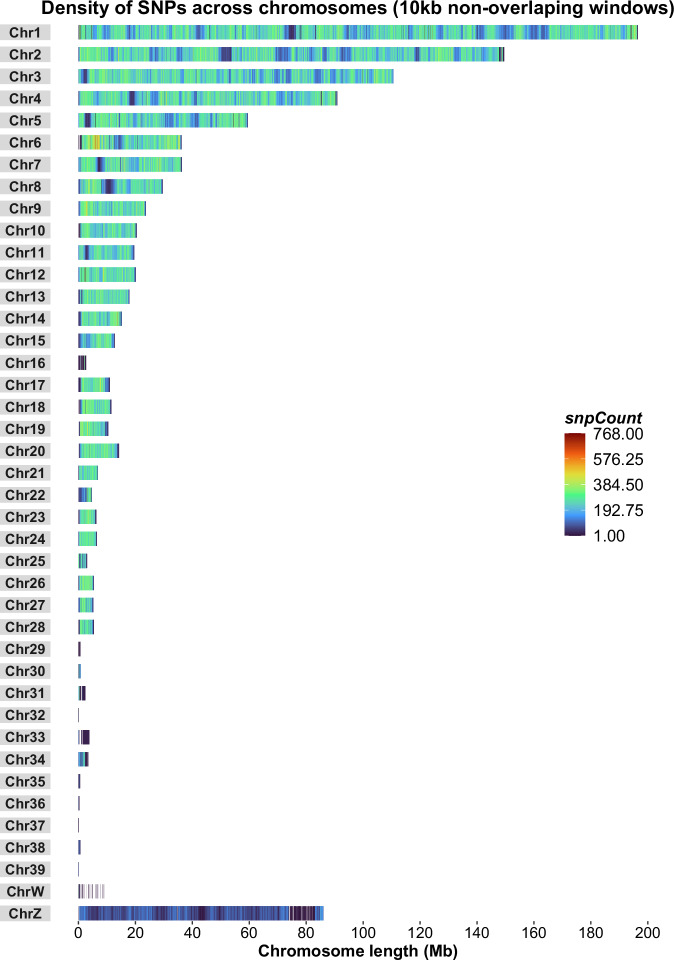
Chromosome-wise SNP distribution heat map across the Bangladeshi native chicken genome based on more than 22 M identified SNPs. The x-axis represents the chromosome length (Mb) and the y-axis denotes chromosome number.

We observed an average of 1 SNP in every 57 base pairs in 10 kb non-overlapping windows across the studied genome. Indigenous chicken species exhibit higher SNP density mainly due to their greater genetic diversity, exposure to varied environmental conditions and more natural settings or less controlled breeding compared to the commercial breeds^53,56,57^. The SNP density across various chromosomes including the sex chromosomes (Z and W) from our study is detailed in Supplementary Table 3 and illustrated in Fig. 4.

## Supplementary information

Supplementary Information

## Data Availability

WGS data analyses from 96 Bangladeshi indigenous chickens were performed using standard bioinformatic tools in the Scientific Linux 7-based High Performance Computing (HPC) system (Eddie) of the University of Edinburgh. The codes used along with versions and parameters of the primary software/tools are available on GitHub (https://github.com/MAGRabbani/WGS_of_BDchicken_data_analysis_codes) and also included in the supplementary materials.
