# Supplementary material for: Whole genome sequencing of three native chicken varieties (Common Deshi, Hilly and Naked Neck) of Bangladesh
Source: Sci Data. 2024 Dec 24;11:1432. doi: 10.1038/s41597-024-04291-z (PMC11668823; doi:10.1038/s41597-024-04291-z)
Supplement: Supplementary file 1 — Supplementary Information [file 41597_2024_4291_MOESM1_ESM.pdf]

Supplementary Information

Supplementary Tables 2

Supplementary Table 1: Sampling details of BLRI-improved chickens and unimproved native chickens 2

Supplementary Table 2: Details of SNPs in different annotation categories according to improved and unimproved Bangladeshi native chicken populations 3

Supplementary Table 3. Summary statistics of SNP density for each chromosome 4

Code and pipeline details 5

Step_1: Quality control of FASTQ files (FastQC-v0.11.7) 5

Step_2: Mapping/Alignment of raw reads against reference genome 5

Step_3: BQSR using GATK and Picard tools 6

Step_4: Variant calling using GATK Call variants in gVCF mode for cohort analysis (GATK-v4.0.10.1) 7

Step_5: Joint genotyping of a cohort of samples 8

Step_6: VQSR 8

Step_7: Select Variants (GATK-v4.0.10.1) 9

Step_8: Filtration of variants (VCFtools-v0.1.13) 9

Supplementary Tables

Supplementary Table 1: Sampling details of BLRI-improved chickens and unimproved native chickens

Population Type	Chicken Variety	Location	Number of samples		
			**Male**	**Female**	**Total**
**Improved**	Common Deshi (CD)	BLRI HQ*, Savar, Dhaka	16	57	73
	Hilly (HL)	BLRI HQ, Savar, Dhaka	15	58	73
	Naked Neck (NN)	BLRI HQ, Savar, Dhaka	15	54	69
	**A. BLRI-Improved sub-total =**		**46**	**169**	**215**
**Unimproved**	Common Deshi (CD)	Monirampur, Jessore	1	4	5
		Dhamrai, Dhaka	8	43	51
		Sadar, Mymensingh	1	4	5
		Bhaluka, Mymensingh	1	4	5
		Chhagalnaiya, Feni	2	6	8
		Gopalpur, Tangail	1	2	3
		Sadar, Gaibandha	2	4	6
	**a. Sub-total of Unimproved CD =**		**16**	**67**	**83**
	Hilly (HL)	Naikhongchhari, Bandarban	5	28	33
		BLRI Regional Research Farm, Naikhongchhari, Bandarban	3	7	10
	**b. Sub-total of Unimproved HL =**		**8**	**35**	**43**
	Naked Neck (NN)	Dhamrai, Dhaka	2	6	8
		Naikhongchhari, Bandarban	2	5	7
		Sadar, Mymensingh	0	5	5
		Bhaluka, Mymensingh	3	8	11
		Gopalpur, Tangail	3	15	18
	**c. Sub-total of Unimproved NN =**		**10**	**39**	**49**
	**B. Unimproved sub-total (a+b+c) =**		**34**	**141**	**175**
**Total (A+B) =**			**80**	**310**	**390**

*Poultry Research Farm, BLRI Headquarters, Savar, Dhaka-1341, Bangladesh;

CD=Common Deshi, HL=Hilly, NN=Naked Neck, Imp=BLRI-Improved native chicken and Unimp=Unimproved native chickens from different localities of Bangladesh

Supplementary Table 2: Details of SNPs in different annotation categories according to improved and unimproved Bangladeshi native chicken populations

Category of Variants		Chicken Populations					
		**CD_Imp**	**CD_Unimp**	**HL_Imp**	**HL_Unimp**	**NN_Imp**	**NN_Unimp**
Exonic	Synonymous	148,881	177,139	140,733	163,393	119,984	175,499
	Missense	79,103	97,995	73,884	88,108	61,717	97,000
	Stop-gained	1,145	1,395	1,068	1,284	901	1,371
	Stop-lost	154	187	157	174	135	178
	Start-lost	716	832	683	784	603	843
	Stop retained	108	112	103	118	81	124
	Coding sequence	1	0	1	1	0	0
Splicing	Splice acceptor	1,201	1,440	1,116	1,278	982	1,411
	Splice donor	1,709	2,055	1,598	1,848	1,351	2,018
	Splice donor 5th base	2,009	2,385	1,909	2,194	1,610	2,377
	Splice region	30,541	36,125	29,027	33,396	24,915	35,900
	Splice donor region	6,347	7,455	5,984	6,937	5,134	7,471
	Splice polypyrimidine tract	25,953	30,656	24,594	28,342	21,188	30,483
Intronic	Intron	9,989,055	11,725,889	9,451,786	10,868,915	8,172,098	11,658,148
Intergenic	Intergenic	1,933,654	2,274,785	1,818,241	2,098,964	1,554,324	2,257,038
Regulatory	Upstream gene	889,060	1,042,641	841,504	966,442	725,724	1,037,953
	Downstream gene	551,657	646,614	523,798	600,319	449,811	644,759
UTR	5 prime UTR	92,561	109,872	87,578	101,342	74,758	109,093
	3 prime UTR	302,386	359,573	286,653	332,936	244,623	357,871
Others	Mature miRNA	116	162	116	153	83	149
	Non-coding transcript exon	528,953	625,312	501,537	576,305	429,858	621,614
		**14,585,310**	**17,142,624**	**13,792,070**	**15,873,233**	**11,889,880**	**17,041,300**

CD=Common Deshi, HL=Hilly, NN=Naked Neck, Imp=BLRI Improved native chickens and Unimp=Unimproved native chickens from different localities of Bangladesh

Supplementary Table 3. Summary statistics of SNP density for each chromosome

Chromosome	Ensemble accession of chromosome	Total SNPs count	SNP density/KB
1	CM028482.1	4549449	23.161
2	CM028483.1	3434599	22.9718
3	CM028484.1	2622118	23.7025
4	CM028485.1	2180914	24.0028
5	CM028486.1	1400582	23.5443
6	CM028487.1	963457	26.6067
7	CM028488.1	898521	24.7159
8	CM028489.1	668611	22.6226
9	CM028490.1	619777	26.1609
10	CM028491.1	502562	24.5848
11	CM028492.1	437352	22.3367
12	CM028493.1	517516	25.7496
13	CM028494.1	453999	25.3588
14	CM028495.1	380810	24.9156
15	CM028496.1	287895	22.6707
16	CM028497.1	9808	3.68306
17	CM028498.1	262922	23.7809
18	CM028499.1	288032	25.0812
19	CM028500.1	253391	24.3786
20	CM028501.1	353961	24.9743
21	CM028502.1	172584	25.1911
22	CM028503.1	71068	15.2408
23	CM028504.1	154058	24.7881
24	CM028505.1	168827	26.3052
25	CM028506.1	53683	17.5034
26	CM028507.1	145033	27.1496
27	CM028508.1	114756	22.0303
28	CM028509.1	117867	21.9124
29	CM028510.1	587	0.823282
30	CM028511.1	10001	13.4422
31	CM028512.1	13328	5.56725
32	CM028513.1	624	6.78261
33	CM028514.1	5753	1.50958
34	CM028515.1	39243	11.3748
35	CM028514.1	2853	5.28333
36	CM028517.1	877	2.53468
37	CM028518.1	70	0.569106
38	CM028519.1	4794	7.18741
39	CM028520.1	1633	9.54971
W	CM028521.1	1976	0.217621
Z	CM028522.1	588957	6.84539

Code and pipeline details

The codes, along with versions and parameters of the primary software/tools used, are provided below and also available on GitHub (https://github.com/MAGRabbani/WGS_of_BDchicken_data_analysis_codes).

Step_1: Quality control of FASTQ files (FastQC-v0.11.7)

```
fastqc -t 1 ${READs}.fastq.gz -o ${READs}
```

Step_2: Mapping/Alignment of raw reads against reference genome

a. Mapping reads with BWA (bwa-v0.7.15)

```
bwa mem -t 4 -M -R "@RG\tID:${SAMPLE}\tSM:${SAMPLE}\tPL:DNBSEQ\tLB:${S
AMPLE}\tPU:unkn-0.0" ${REF_GENOME} ${READS_1} ${READS_2} > ${SAMPLE}.sam
```

b. Sort SAM into coordinated order and save as BAM (picard-v2.25.4)

```
picard SortSam \
    I=${SAMPLE}.sam \
    O=${SAMPLE}_sorted.bam \
    SORT_ORDER=coordinate \
    TMP_DIR= tmp_${SAMPLE}
```

c. Mark duplicates and create bam index (picard-v2.25.4)

```
picard MarkDuplicates \
    I=${SAMPLE}_sorted.bam \
    O=${SAMPLE}_mdup.bam \
    CREATE_INDEX=true \
    M=metrics/${SAMPLE}_mdup_metrics.txt \
    TMP_DIR=tmp_${SAMPLE} \
    MAX_FILE_HANDLES_FOR_READ_ENDS_MAP=4000 \
    OPTICAL_DUPLICATE_PIXEL_DISTANCE=2500
```

d. Validate BAM file (picard-v2.25.4)

```
picard ValidateSamFile \
    I=${SAMPLE}_mdup.bam \
    MODE=SUMMARY \
    MAX_OPEN_TEMP_FILES=8000 \
    TMP_DIR=tmp_${SAMPLE}
```

e. Flagstat of BAM file (samtools-v1.13)

```
samtools flagstat ${SAMPLE}_sorted.bam
samtools flagstat ${SAMPLE}_mdup.bam
```

Step_3: BQSR using GATK and Picard tools

a. Analyze of the patterns of covariation in the sequence dataset (GATK-v4.0.10.1)

```
gatk BaseRecalibrator \
    -R ${REF_GENOME} \
    -I ${SAMPLE}_mdup.bam \
    --known-sites ${VCF} \
    --output ${SAMPLE}_recal_data.table
```

b. A second pass to analyze covariation post-recalibration (GATK-v4.0.10.1)

```
gatk BaseRecalibrator \
    -R ${REF_GENOME} \
    -I {SAMPLE}_mdup.bam \
    --known-sites ${VCF} \
    --output ${SAMPLE}_post_recal_data.table
```

c. Generate before/after plots (GATK-v4.0.10.1)

```
gatk AnalyzeCovariates \
    -before ${SAMPLE}_recal_data.table \
    -after ${SAMPLE}_post_recal_data.table \
    -plots ${SAMPLE}_recalibration_plots.pdf
```

d. Apply the recalibration to your sequence data (GATK-v4.0.10.1)

```
gatk PrintReads \
    -R ${REF_GENOME} \
    -I ${SAMPLE}_mdup.bam \
    -O ${SAMPLE}_recal.bam
```

e. Validate the recalibrated BAM file (picard-v2.25.4)

```
picard ValidateSamFile \
    I=${SAMPLE}_recal.bam \
    MODE=SUMMARY \
    TMP_DIR=tmp_${SAMPLE} \
    MAX_OPEN_TEMP_FILES=4000
```

f. Flagstat of recalibrated BAM file (samtools-v1.13)

```
samtools flagstat ${SAMPLE}_recal.bam
```

g. Additional step: Insert Size metrics (picard-v2.25.4)

```
picard CollectInsertSizeMetrics \
    I=${SAMPLE}_mdup.bam \
    O=${SAMPLE}_mdup_insertSize_metrics.txt \
    HISTOGRAM_FILE=${SAMPLE}_mdup_insertSize_metrics.pdf
```

Step_4: Variant calling using GATK Call variants in gVCF mode for cohort analysis (GATK-v4.0.10.1)

```
gatk HaplotypeCaller \
    -R ${REF_GENOME} \
    -I ${SAMPLE}_recal.bam \
    -O ${SAMPLE}.g.vcf.gz \
    -ERC GVCF
```

Step_5: Joint genotyping of a cohort of samples

a. GenomicsDBImport (GATK-v4.0.10.1):

```
gatk GenomicsDBImport \
    --genomicsdb-workspace-path my_database \
    --intervals interval.bed \
    -V ${SAMPLE}1.g.vcf.gz \
    -V ${SAMPLE}3.g.vcf.gz \
    -V ${SAMPLE}3.g.vcf.gz
```

b. GenotypeGVCFs (GATK-v4.0.10.1)

```
gatk GenotypeGVCFs \
    -R ${REF_GENOME} \
    -V gendb://my_database \
    -O ${SAMPLE}.vcf.gz
```

Step_6: VQSR

a. VariantRecalibrator (GATK-v4.0.10.1)

```
gatk VariantRecalibrator \
    -R ${REF_GENOME} \
    -V ${SAMPLE}.vcf.gz \
    --resource GRCg7b_dbSNP,known=true,training=false,truth=false,pr
ior=2.0:${KNOWNVAR} \
    --resource GCRg6a_validated_snp,known=false,training=true,truth=
true,prior=12.0:${TRUEVAR} \
    -an DP -an QD -an MQRankSum -an ReadPosRankSum -an FS -an SOR \
    -mode SNP \
    -tranche 100.0 -tranche 99.9 -tranche 99.0 -tranche 90.0 \
    -O ${SAMPLE}.SNPs.recal.gz \
    --tranches-file ${SAMPLE}.SNPs.tranches \
    --rscript-file ${SAMPLE}_recalSNPS.plots.R
```

b. ApplyVQSR (GATK-v4.0.10.1)

```
gatk ApplyVQSR \
    -R ${REF_GENOME} \
    -V ${SAMPLE}.vcf.gz \
    --mode SNP \
    --truth-sensitivity-filter-level 99.0 \
    --recal-file ${SAMPLE}.SNPs.recal.gz \
    --tranches-file${SAMPLE}.SNPs.tranches \
    -O ${SAMPLE}_recalSNPs.vcf.gz
```

Step_7: Select Variants (GATK-v4.0.10.1)

```
gatk SelectVariants \
    -R ${REF_GENOME}\
    -V ${SAMPLE}_recalSNPs.vcf.gz \
    --select-type SNP \
    --restrict-alleles-to BIALLELIC \
    -O ${SAMPLE}.vcf.gz
```

Step_8: Filtration of variants (VCFtools-v0.1.13)

```
vcftools \
    --gzvcf ${SAMPLE}.vcf.gz \
    --hwe 0.00001 \
    --max-missing 0.9 \
    --minGQ 20.0 \
    --minDP 3 \
    --min-alleles 2 \
    --max-alleles 2 \
    --recode \
    --recode-INFO-all \
    --out ${SAMPLE}_fil.vcf.gz
```
